# Status epilepticus in the Canadian Arctic: A public health imperative hidden in plain sight

**DOI:** 10.1002/epi4.12538

**Published:** 2021-09-18

**Authors:** Marcus C. Ng, Milena Pavlova

**Affiliations:** ^1^ Section of Neurology University of Manitoba Winnipeg Manitoba Canada; ^2^ Department of Neurology Brigham and Women’s Hospital Harvard Medical School Boston Massachusetts USA

**Keywords:** epilepsy, health care inequity, Nunavut, seizures, underserved population

## Abstract

**Objective:**

The World Health Organization, International League Against Epilepsy (ILAE), and International Bureau for Epilepsy have called epilepsy a public health imperative, with appropriate emphasis on low‐to‐middle‐income countries (LMIC). Although Canada is a high‐income country (HIC), income is not distributed uniformly. Furthermore, epilepsy data from the national statistical agency explicitly overlook the Arctic by excluding these territories. A common neurologic emergency, status epilepticus (SE) is a life‐threatening manifestation of epilepsy that demands prompt treatment to avoid death and long‐term sequelae. Therefore, we examined the rate of SE in a well‐defined Canadian Arctic region.

**Methods:**

This study takes epidemiologic advantage of the Kivalliq Region's geographical isolation, which is accessible only by air. All SE patients requiring emergency care are consistently flown 1200‐1900 kilometers to a single designated hospital in a distinct southern part of Canada for further management and electroencephalography (EEG). We conducted a retrospective database and chart review at this “bottleneck” hospital to identify patients with seizure(s) severe enough to justify emergency airborne medical evacuation over a 11.25‐year period from 2009 to 2020.

**Results:**

We screened 40 392 EEGs to yield 117 distinct medical evacuations for “operational SE” from 99 patients to derive estimated SE incidences of 99.9 evacuations per 100 000/year and 84.5 patients per 100 000/year. The average time from seizure onset to EEG was 3.2 days. Only 16.2% of SE patients had known epilepsy. For “confirmed SE” cases meeting ILAE criteria, or cases with persistently epileptiform EEG despite days of empiric treatment, estimated incidence was 77.7 evacuations per 100 000/year and 64.9 patients per 100 000/year.

**Significance:**

High SE and epilepsy rates in the Canadian Arctic are consistent with LMIC rather than HIC. Our findings demonstrate the paradox of LMIC‐equivalent epilepsy populations camouflaged within HIC. Our findings also highlight the long‐standing plight of these under‐served and overlooked populations hidden in plain sight.

**Key Points**

- Patients with uncontrollable seizures in Kivalliq, Nunavut, Canada, are evacuated by air to a single site over 1200‐1900 km.
- EEG and chart review at the “bottleneck” evacuation site were performed over 11.25 years using ILAE status epilepticus criteria.
- Incidences of operational and confirmed status epilepticus are at least 99.9 and 77.7 evacuations per 100  000/year, respectively.
- Epilepsy care resources in both the Canadian Arctic, and the non‐Arctic evacuation site, are very limited.
- High rates of status epilepticus and epilepsy in the Canadian Arctic are consistent with low‐to‐medium‐income countries.

## 1 INTRODUCTION

**FIGURE 1:**
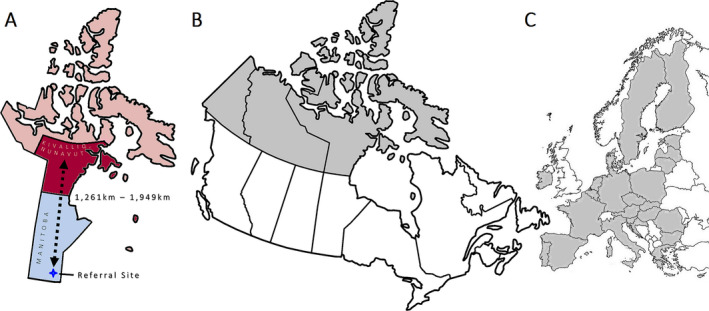
(A) SE patients from the Kivalliq Region (deep red) of the northern Canadian Arctic territory of Nunavut (light red) are medically evacuated to the nearest designated neurosciences hospital (deep blue star) in the adjacent southern province of Manitoba (light blue) for further assessment and electroencephalography. (B) Outline map of Canada (thick lines) with the three Canadian Arctic territories shaded. (C) Juxtaposed outline map of Europe (thin lines) with the member states of the European Union (EU) shaded. The Canadian Arctic territories are comparable in size to the entire land mass of the EU [maps not to scale]

The Canadian Arctic territories alone are almost 4 million square kilometers in area, which is comparable in size to the entire European Union combined (Figure 1).^1^, ^2^ However, these territories are sparsely populated with some of the lowest population densities on Earth.^1^ Delivery of modern medicine remains challenging, even though these Arctic territories are technically located within a high‐income country (HIC). Furthermore, health care does not fall under national federal jurisdiction in Canada. Instead, each individual southern province delivers an arbitrary patchwork of services that may or may not include the northern Arctic territories.^3^ This non‐standardized healthcare infrastructure often leads to widespread geographical disparities across the second largest country on the planet.

Such challenges can impact the delivery of health care for epilepsy and seizures. Epilepsy affects at least 50 million people worldwide with three times the risk of premature death.^4^ The International League Against Epilepsy (ILAE), International Bureau for Epilepsy, and World Health Organization (WHO) have recently designated epilepsy as a public health imperative.^4^ The WHO also notes that epilepsy tends to affect more people in low‐to‐middle‐income countries (LMIC).^4^ However, income distribution is often unequal in both LMIC and HIC. For example, extraordinarily wealthy persons in LMIC can have more favorable social determinants of health than the most impoverished persons in a HIC. While Canada is overall high in the United Nations Human Development Index, its Arctic territory of Nunavut would rank 45th globally as its own jurisdiction.^5^ Furthermore, official policy‐informing national government data often entirely ignore the Arctic population.^6^

Similarly, healthcare delivery challenges and unfavorable social determinants of health can affect the frequency and severity of seizures, including status epilepticus (SE). Because SE is a common and life‐threatening neurologic emergency that demands prompt treatment to avoid death and long‐term sequelae, we sought to determine rates of SE in the Canadian Arctic for the very first time. Specifically, we examine a vast, isolated, but well‐defined, northern geographical region accessible only by air^1^ and serviced exclusively by an academic medical center in a distinct southern part of the country.

## 2 METHODS

The University of Manitoba Research Ethics Board approved this study. The designated neurosciences hospital for the province of Manitoba (“referral site”) provides specialty healthcare services for the Kivalliq Region (“Region”) of the Canadian Arctic territory of Nunavut. This territory was created in 1999 and is the country's newest (Figure 1). Each isolated community within the Region is accessible only by air. Such isolation effectively isolates settlements into functional islands even if they are situated on the mainland.^1^ All patients with clinically diagnosed uncontrollable seizures require airborne medical evacuation for further management. They are empirically treated for SE before being flown a distance of approximately 1200‐1900 km^1^ over many hours to days to a single referral site in Winnipeg, Manitoba. At this referral site, patients undergo further clinical assessment and electroencephalography (EEG) to rule out ongoing electrographic seizures.

We searched the referral site's electronic EEG database for inpatient tests with a registered Nunavut healthcare insurance number. The search start date was September 2009 when EEG tests transitioned from paper to computer. The search end date was November 2020 when revised COVID‐19 policy curtailed referral EEG volume. Exclusion criteria were age under 16 years, repeat EEGs during the same evacuation, patients flown to the referral site for a routinely scheduled EEG appointment or elective epilepsy monitoring, patients who developed SE outside the Kivalliq Region while at the referral site awaiting an unrelated elective medical service, and patients with documented sole diagnosis of psychogenic nonepileptic seizures (PNES).

All included EEGs were designated as clinically “operational SE.” We then reviewed both paper and electronic medical records, including neuroimaging databases, to further refine SE diagnoses into cases with (a) characteristics meeting the official ILAE definition of SE based on clinical criteria alone,^7^ (b) an epileptiform EEG, (c) abnormal but non‐epileptiform EEG, and (d) unremarkable EEG. We classified the seizure(s) prompting medical evacuation as generalized motor, focal motor, focal non‐motor, paroxysmal loss of consciousness (LOC), or continuous LOC. Epileptiform EEGs included evidence of ongoing SE (defined by the Salzburg criteria^8^), burst suppression, patterns along the interictal–ictal continuum, and sporadic epileptiform discharges. Abnormal but non‐epileptiform EEGs showed focal or diffuse slowing. Unremarkable EEGs did not show definite abnormality, only showed medication effect from empiric SE treatment, or yielded recording artifact. We used Stata 14 software to calculate and manage descriptive statistics (College Station, USA).

## 3 RESULTS

**FIGURE 2:**
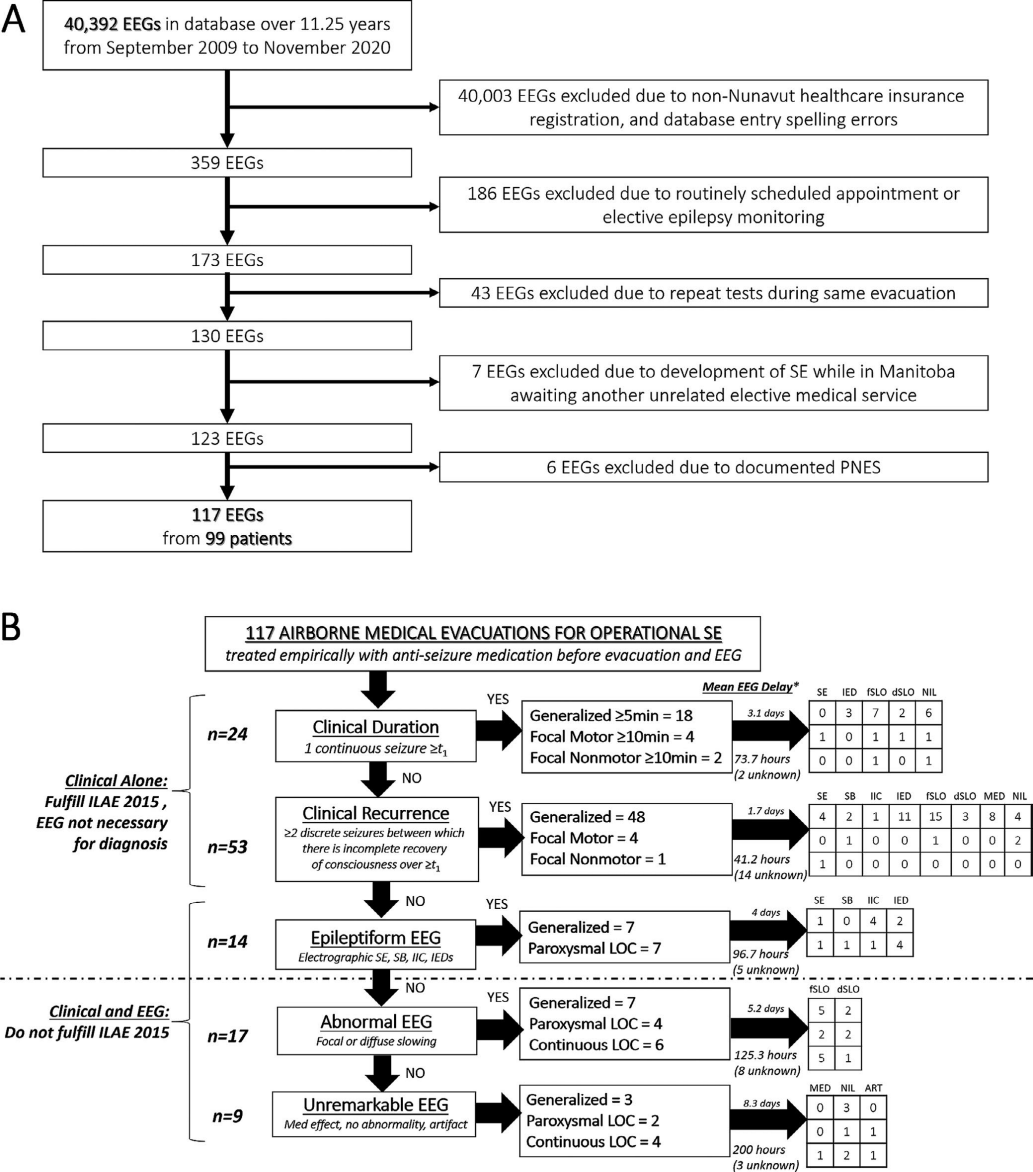
(A) Electroencephalogram (EEG) and patient selection flow diagram. (B) Definitions of status epilepticus (SE) among included EEGs. ART, artifact; dSLO, diffuse slowing; fSLO, focal slowing; IED, interictal epileptiform discharges; IIC, interictal‐ictal continuum; ILAE, International League Against Epilepsy; LOC, loss of consciousness, MED, medication effect; PNES, psychogenic nonepileptic seizures; SB, burst suppression

We found 40 392 EEG tests over 11.25 years at the referral site's EEG laboratory. We included 117 EEG tests after applying exclusion criteria (Figure 2A). These tests represented 117 distinct medical evacuations for clinically “operational SE” from 99 persons to yield 1.18 evacuations per patient with a registered Nunavut healthcare insurance number. 56% of patients were male. Mean age for all patients was 41.2 years (range 16‐84, Table 1). We used the age at time of first evacuation when a patient was evacuated multiple times. In eight patients (8%), operational SE started in sleep. Evacuations were most commonly attributable to self‐harm or substance use, followed by no known cause (idiopathic), history of epilepsy, hemorrhagic stroke, and systemic illness.

**TABLE 1:** Status epilepticus characteristics of the Kivalliq Region (2009‐2020)

Age		Etiology						EEG test location			
Years	n	#	Per evacuation	%	#	Per capita	%	#		Per evacuation (%)	Per capita (%)
16‐20	13	1	Self‐Harm / Substances	22.2	1	Idiopathic	22.2	1	ED	59.8	60.6
21‐30	24	2	Idiopathic	20.5	2	Self‐Harm/Substances	21.2	2	Ward^d^	17.9	16.2
31‐40	13	3	Epilepsy	18.8	3	Hemorrhagic Stroke	20.2	3	ICU	14.5	14.1
41‐50	17	4	Hemorrhagic Stroke	16.2	4	Systemic Illness^a^	19.2	4	Other^e^	7.7	9.1
51‐60	15	5	Systemic Illness^a^	15.4	5	Epilepsy	16.2				
61‐70	6	6	ASM Nonadherence	14.5	6	Ischemic Stroke	14.1				
71‐80	10	7	Ischemic Stroke	12	6	Traumatic	14.1				
81‐84	1	8	Traumatic	11.1	8	Metabolic^b^	12.1				
		9	Metabolic^b^	10.3	9	ASM Nonadherence	11.1				
		10	Cerebral Infection	3.4	10	Cerebral Infection	4				
		10	Pregnancy	3.4	10	Pregnancy	4				
		12	Genetic^c^	2.6	12	Genetic^c^	2				
		13	Radiation Necrosis	1.7	13	Radiation Necrosis	1				

Note

The sum of all percentages exceeds 100%, as patients were often evacuated for operational SE attributable to more than one etiology.

Abbreviation: #, rank; ASM, antiseizure medication; ED, emergency department; EEG, electroencephalogram; ICU, intensive care unit.

^a^ Multi‐organ failure, sepsis, tuberculosis, pneumonia, asthma, sickle cell anemia, vasculitis, lupus.

^b^ Hyponatremia, hypoglycemia, hypocalcemia.

^c^ CADASIL, PARK2 mutation of unknown significance.

^d^ Internal medicine ward.

^e^ High observation unit, stepdown unit, or neurosurgical ward.

The “self‐harm or substance use” category fell from first to second place when etiologies were re‐sorted from a per‐evacuation to a per‐capita basis because some patients were repeatedly evacuated. Similarly, “antiseizure medication nonadherence” fell from sixth to ninth place because of repeated evacuations for this provoking factor. At least one instance of nonadherence leading to operational SE was the reported direct result of the only pharmacy in the community running out of supply due to a blizzard affecting shipment.

Ninety‐six patients had neuroimaging after an evacuation. 75.8% of all neuroimaging were MRI scans. 58.3% of all neuroimaging was abnormal. The most specific epileptogenic lesions occurred in 10 patients with hippocampal or mesial temporal changes on MRI. Only 2 of these 10 patients had a documented history of epilepsy throughout the 11.25‐year period. Only 1 patient underwent epilepsy surgery in 2011, which was one year before Manitoba could no longer sustain intracranial EEG capability. Overall, only 16.2% of 99 patients had a known history of epilepsy who accounted for 18.8% of 117 evacuations.

The average time from symptom onset in the Kivalliq Region to EEG recording at the referral site was 3.2 days (Figure 2B), when such data were available on chart review (72.6% of 117 EEGs). Inpatient EEG testing at the referral site most often occurred in the Emergency Department, followed by an internal medicine ward, an intensive care unit, and finally a high observation unit, stepdown unit, or neurosurgical ward (the referral site does not have a dedicated geographical specialty neurology ward). Most evacuations (70.9%) were due to generalized motor SE.

Seventy‐seven of 117 (65.8%) evacuations met the ILAE definition for generalized motor, focal motor, and focal non‐motor SE based on clinical grounds alone. Empiric treatment started 2.2 mean days (53 hours) before EEG. Six of 77 (7.8%) evacuations still demonstrated ongoing electrographic SE. Most other EEGs showed focal slowing (32.5%) at the time of recording. An additional 14 of 117 (12%) evacuations did not meet ILAE criteria, but demonstrated epileptiform activity on EEG starting 4 mean days (96.7 hours) after empiric treatment had begun. Two of these 14 (14.3%) additional cases showed ongoing electrographic SE. Another 17 of 117 (14.5%) EEGs were abnormal but non‐epileptiform by the time recording started 5.2 average days (125.3 hours) later. Nine more of 117 (7.7%) EEGs were unremarkable by the time recording began a mean 8.3 days (200 hours) later.

The most recent 2016 national government census population data for the Kivalliq Region was 10 413.^9^ A total of 117 medical evacuations from this population represented 10.4 operational SE cases per year and 8.8 distinct persons undergoing medical evacuations for operational SE per year. This translates into an estimated incidence of 99.9 evacuations per 100 000/year and 84.5 patients per 100 000/year. We can further define “confirmed SE” as ILAE‐confirmed SE or operational SE cases with a demonstrably persistent epileptiform EEG. Rates of “confirmed SE” cases are 77.7 evacuations per 100 000/year and 64.9 patients per 100 000/year.

## 4 DISCUSSION

**FIGURE 3:**
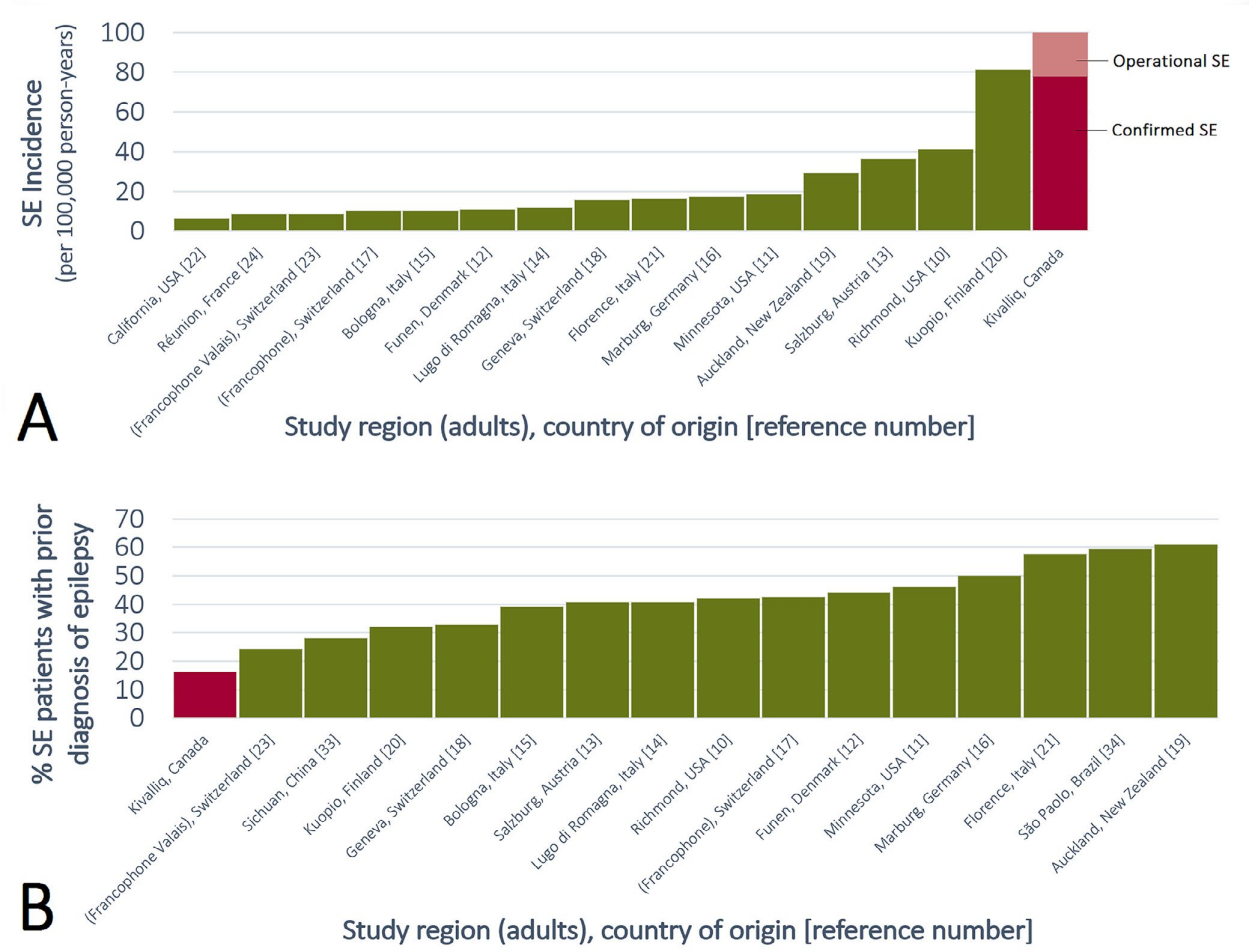
(A) Reported status epilepticus (SE) incidence for adults in the global literature. (B) Distribution of adult SE patients with known prior diagnosis of epilepsy as reported in the global literature. The median of 41% is in agreement with a seminal SE study by DeLorenzo et al.^10^ Note the over‐representation of high‐income countries

Despite a relatively low reported history of epilepsy, we found a strikingly high estimated incidence of SE in the Kivalliq Region of the Canadian Arctic territory of Nunavut. Our “operational SE” incidences of 99.9 evacuations per 100 000/year and 84.5 patients per 100 000/year rank high in the global SE literature (Figure 3A, Table 2).^10^, ^11^, ^12^, ^13^, ^14^, ^15^, ^16^, ^17^, ^18^, ^19^, ^20^, ^21^, ^22^, ^23^, ^24^ Although high SE incidences have also been reported for pediatric or elderly subgroups, they represent the known bimodal epidemiologic peaks of SE.^10^, ^11^ In contrast, our study excluded the first pediatric peak because patients were 16 years of age or over. The majority of our SE cohort was well within middle age (mean 41.2 years), which usually represents an incidence nadir. Even after applying more stringent criteria to only consider “confirmed SE” cases, our estimated incidence rates of 77.7 evacuations per 100 000/year and 64.9 patients per 100 000/year still remain elevated.^10^, ^11^, ^12^, ^13^, ^14^, ^15^, ^16^, ^17^, ^18^, ^19^, ^20^, ^21^, ^22^, ^23^, ^24^ Unfortunately, global literature of SE incidence is biased toward HIC due to a paucity of studies from LMIC, especially on adult patients. However, SE generally occurs more often in LMIC than HIC. For example, a district hospital‐based cohort study of pediatric convulsive SE from rural Kenya reported incidences as high as 268 cases per 100 000/year in the 1‐11 month age group.^25^, ^26^

**TABLE 2:** Adult global literature of reported status epilepticus (SE) incidence and proportions of adult SE patients with a known history of epilepsy

References	Locale/region, country	Years	SE definition	Ages	Study design	SE incidence (/100 000 person‐years)	% SE with prior epilepsy diagnosis
Hesdorrfer et al.	Minnesota, USA	1965‐1984	30 min^a^	All	Retrospective	18.3^d^	46
DeLorenzo et al.	Richmond/Virginia, USA	1989‐1991	30 min^a^	All	Prospective	41	42
Garzon et al.	São Paolo, Brazil	1989‐1993	ILAE 1981, 30 min^a^	≥3 mo	Retrospective	n/a	59.4
Wu et al.	California, USA	1991‐1998	Generalized SE	All	Retrospective	6.2	n/a
Li et al.	Sichuan, China	1996‐2007	30 min^a^	≥15 y	Prospective	n/a	28.1^b^
Coeytaux et al.	French‐speaking Switzerland	1997‐1998	30 min^a^, non‐anoxic	All	Prospective	10.3^d^	42.4
Jallon et al.	Geneva, Switzerland	1997‐1998	30 min^a^, non‐anoxic	All	Prospective	15.5	32.8
Knake et al.	Marburg, Germany	1997‐1999	30 min^a^	≥18 y	Prospective	17.1^d^ ^,^ ^c^	50
Vignatelli et al. (2003)	Bologna/Emilia‐Romagna, Italy	1999‐2000	30 min^a^	≥20 y	Prospective	10.7^d^	39
Vignatelli et al. (2005)	Lugo di Romagna/Ravenna/Emilia‐Romagna, Italy	1999‐2001	30 min^a^	≥20 y	Prospective	11.6^e^	40.7
Bhalla et al.	Réunion, France	2004‐2005	30 min^a^	All	Prospective	8.52	excluded
Rodin et al.	Funen, Denmark	2008‐2013	5 min, first‐time, non‐anoxic	≥18 y	Prospective	10.7	43.9
Kantanen et al.	Kuopio, Finland	2010‐2012	ILAE 2015	≥16 y	Prospective	81^c^	32
Leitinger et al.	Salzburg, Austria	2011‐2015	ILAE 2015, non‐anoxic	≥18 y	Retrospective	36.1^d^	40.7
Bergin et al.	Auckland, New Zealand	2015‐2016	ILAE 2015, except convulsive episodes ≥10 min	All	Prospective	29.25^c^, ^d^	61
Nazerian et al.	Florence, Italy	2016	ILAE 2015	≥18 y	Retrospective	16^c^	57.6
Vijiala and Alvarez	French‐speaking Valais, Switzerland	2015‐2019	ILAE 2015	≥17 y	Prospective	8.6	24.3
Ng and Pavlova	Kivalliq/Nunavut, Canada	2009‐2020	ILAE 2015 and EEG; [operational]	≥16 y	Retrospective	77.7^c^ [99.9^c^]	16.2

Abbreviation: ILAE, International League Against Epilepsy.

^a^ Any seizure lasting for 30 min or longer, or intermittent seizures lasting for greater than 30 min from which the patient did not regain consciousness.

^b^ Of the patients who died (n = 32).

^c^ Incidence of SE episodes.

^d^ Age‐adjusted incidence.

^e^ Age‐ and sex‐adjusted incidence.

Given the Kivalliq Region's high estimated SE incidences for a HIC, we believe that these rates are consistent with a LMIC rather than a HIC. For example, a male born in Nunavut between 2017 and 2019 can expect to live just 69.3 years.^27^ In comparison, the average life expectancy for Canada in general is 80 and 64.3 years in Kenya.^27^, ^28^ We believe that the discrepancy in life expectancy between Nunavut and the rest of Canada can be explained by social determinants of health, such as the challenges of healthcare delivery. For SE in particular, familiarity with emergency seizure management in the Arctic may be unavoidable due to patients frequently presenting with seizures. However, knowledge of when a seizure becomes SE is likely not widespread because there are no neurologists in the Kivalliq Region. Even at the designated neurosciences hospital to which patients are medically evacuated, resources are very limited for a HIC to serve over 1.5 million people. Specifically, there has been an ongoing neurologist shortage over 11.25 years. There is no stroke unit. Similarly, there is no dedicated geographical specialty neurology ward. Adult epilepsy monitoring occurred idiosyncratically on two beds borrowed from an ill‐equipped orthopedics ward, until their indefinite closure by COVID‐19. There has been no access to intracranial EEG from 2012. Although recurrent self‐harm or substance use was the most common reason for medical evacuation, there is no psychiatric support in the province's only adult epilepsy clinic. Referrals to psychiatry are permissible only by way of a primary care physician.

As a result, formal epilepsy outreach to the Arctic is nonexistent. Most patients are never followed in the epilepsy clinic. At the same time, Indigenous persons comprised 92% of the population in the Kivalliq Region.^1^ A recent study from the adjacent Canadian province of Saskatchewan found that the incidence of epilepsy in the Indigenous population is double the national average.^5^, ^9^, ^10^, ^29^ Although ethnicity data were not available on chart review, there is no reason to assume that Indigenous persons do not comprise a similar majority of SE medical evacuations. Recurrent minor seizures (and by extension, epilepsy) in this population may be under‐reported due to understandable enduring mistrust of the healthcare system resulting from extreme historical inequities,^30^ stigma against seizures, or cultural interpretation of symptoms. The latter can be seen in other conditions. For example, the diagnosis of narcolepsy can be delayed for as long as a decade after symptoms occur.^31^ Like many cultures around the world, unique reports and interpretations of sleep paralysis in the Inuit population stem from a mystical or shamanistic framework.^32^ Similar social considerations may apply to focal seizures that impair consciousness. Reporting may be delayed, and medical attention sought only when seizures become continuous SE.

These factors help explain why only 16.2% of our SE cohort had a reported epilepsy history. Our rates are low compared to the global SE literature. Although biased to HIC, around 40% of SE patients in other studies had a reported history of epilepsy (median 41%, interquartile range 35.5%–43.9%, Figure 3B).^10^, ^11^, ^12^, ^13^, ^14^, ^15^, ^16^, ^17^, ^18^, ^19^, ^20^, ^21^, ^23^, ^33^, ^34^ We believe that this 2.475‐fold discrepancy is due to systemic under‐resourcing and under‐recognition of epilepsy as in LMIC. In contrast, a recent health report from the official government statistical agency cited a national active epilepsy prevalence of 0.41%.^6^ This officially cited rate is consistent with a HIC, but in a calculation that explicitly excludes the Arctic territories.^6^ To account for “missing” unrecognized epilepsy patients, let us assume that HIC resources are available for diagnosis to raise the proportion of patients with an epilepsy history in our SE cohort from the observed LMIC‐compatible 16.2% to the expected 40% from the HIC‐biased literature.^10^, ^11^, ^12^, ^13^, ^14^, ^15^, ^16^, ^17^, ^18^, ^19^, ^20^, ^21^, ^23^, ^33^, ^34^ This yields 24 additional undiagnosed epilepsy patients for a total of 40 SE patients with an epilepsy history. If we presumptuously apply the official active epilepsy prevalence rate of 0.41% to official Kivalliq Region population data, then this yields 43 total persons with epilepsy.^9^ Even against the backdrop of extremely limited health care, the result of 93% (40/43) of all epilepsy patients in the Region experiencing SE within 11.25 years is unlikely. Most persons with epilepsy have discrete seizures that resolve prior to becoming SE.^11^, ^35^ Moreover, as few as 15% of epilepsy patients ever develop SE at all.^11^, ^35^ Rather, it is more likely that there are also many additional undiagnosed epilepsy patients outside of our SE cohort. These additional patients can revise down the total percentage of epilepsy patients with SE from 93% toward a more realistic proportion. However, increasing the total number of epilepsy patients also increases epilepsy prevalence. Accordingly, we can conclude that the prevalence of epilepsy in this region is likely higher than the national 0.41%.

A study limitation is EEG unavailability to confirm SE during emergency treatment in the Region prior to evacuation. By the time patients arrived at the referral site many hours or days later, the first EEG often no longer showed SE after empiric therapy. However, this practice is consistent with SE being a clinical diagnosis demanding immediate clinical management—not EEG, which is an ancillary test.^36^ Furthermore, we believe that the decision made to commit a patient to medical evacuation by air across thousands of kilometers is a rigorous practical threshold that parallels the ILAE clinical time threshold *t*_1_: when treatment should be initiated for SE.^7^ Consequently, not all evacuated patients may have had EEG. However, including these “missing” patients would have only increased our estimated SE incidence rates, which are already among the highest reported in the global SE literature.

An additional limitation is the relatively crude methodology adopted by this study. In the absence of coordinated electronic healthcare infrastructure linking the Arctic with the rest of the country, we relied on databases from the referral site instead of local data directly collected within the Kivalliq Region. In the further absence of coordinated clinical epilepsy care at the referral site, we also relied on the site's EEG database such that “operational SE” became the starting point of review. Because the initial diagnosis of SE is clinical, and patients were already treated prior to evacuation, the data obtained from the referral center should be scrutinized for etiology, predisposing, and contributing factors with caution. It is likely that these factors severely underestimated the actual SE incidence in the Kivalliq Region. At the same time, these limitations represent a tremendous opportunity for future healthcare systems and epidemiology research.

Another study limitation is the northerly location of the Kivalliq Region, which is subject to extreme changes in exposure to light and dark over the course of a year. Although our global literature review was biased to HIC, comparably high SE incidence was also reported from the University of Kuopio catchment area in Finland.^20^ This Finnish area shares the same geographical latitude as the Kivalliq Region, but it does not share the same social determinants of health (eg, HDI, ethnicity).^20^ A possible explanation is that dramatic circadian and circannual changes may disrupt sleep to aggravate SE occurrence in a manner independent of LMIC socioeconomic effects. For example, almost 1 in 10 of our patients had SE witnessed to arise from sleep. Another speculative possibility is that mechanisms terminating a seizure may be altered in Arctic populations. Genetic polymorphisms and altered regulation of metabolic or neuro‐excitatory function may have evolved in adaptation to cold, or long periods of light and dark lasting months. For example, metabolic changes due to genetic causes have been reported for fatty acid desaturases in the unique Arctic environment.^37^ Furthermore, individuals from the Inuit population in three different countries (Alaska, Greenland, and Canada) also have a relatively high rate of obesity, a higher waist circumference among women, higher blood pressure, and lower high‐density lipoproteins.^38^ While these geographical considerations remain speculative and cannot alone account for high SE rates in the Kivalliq Region, they represent another exciting opportunity for further research into the epileptic chronobiology of both Indigenous and non‐Indigenous circumpolar populations.

In summary, SE and epilepsy are endemic to the Canadian Arctic at rates far greater than those officially reported by the national statistical agency for a HIC.^6^ Rather, these rates are consistent with the higher epilepsy burden cited by the WHO for LMIC.^4^ The situation is further aggravated by the nil to few HIC epilepsy care resources at the non‐Arctic referral site to which SE patients are routinely evacuated. To alleviate the suffering of persons with frequent SE in the Kivalliq Region, we echo recent concerted international calls to promote epilepsy as a public health imperative and call for significant investment into epilepsy care. Our findings also demonstrate the paradox that LMIC epilepsy populations around the world do not necessarily need to live within LMIC—they may be part of HIC, hiding in plain sight.

## CONFLICT OF INTEREST

Dr. Ng has nothing to disclose. Dr. Pavlova reports grants from Jazz pharma, personal fees from Sanofi, grants from Lundbeck, grants from Biomobie, personal fees from Massmedical, and personal fees from Oakstone outside the submitted work.

## ETHICAL APPROVAL

We confirm that we have read the Journal's position on issues involved in ethical publication and affirm that this report is consistent with those guidelines.
